# Supplementary material for: An Imidazolium-Based Ionic Liquid as a Model to Study Plasticization Effects on Cationic Polymethacrylate Films
Source: Polymers (Basel). 2023 Feb 28;15(5):1239. doi: 10.3390/polym15051239 (PMC10006978; doi:10.3390/polym15051239)
Supplement: Supplementary file 1 [file polymers-15-01239-s001.zip › polymers-2218886-supplementary.pdf]

An Imidazolium-Based Ionic Liquid as a Green Alternative for Plasticization of Cationic Polymethacrylate Films

Thashree Marimuthu [1], Zainul Sidat [1], Pradeep Kumar [1], and Yahya E. Choonara [1,*]

Supplementary information

Detailed FTIR for pristine polymer film and formulations at 10%w/w of plasticizer

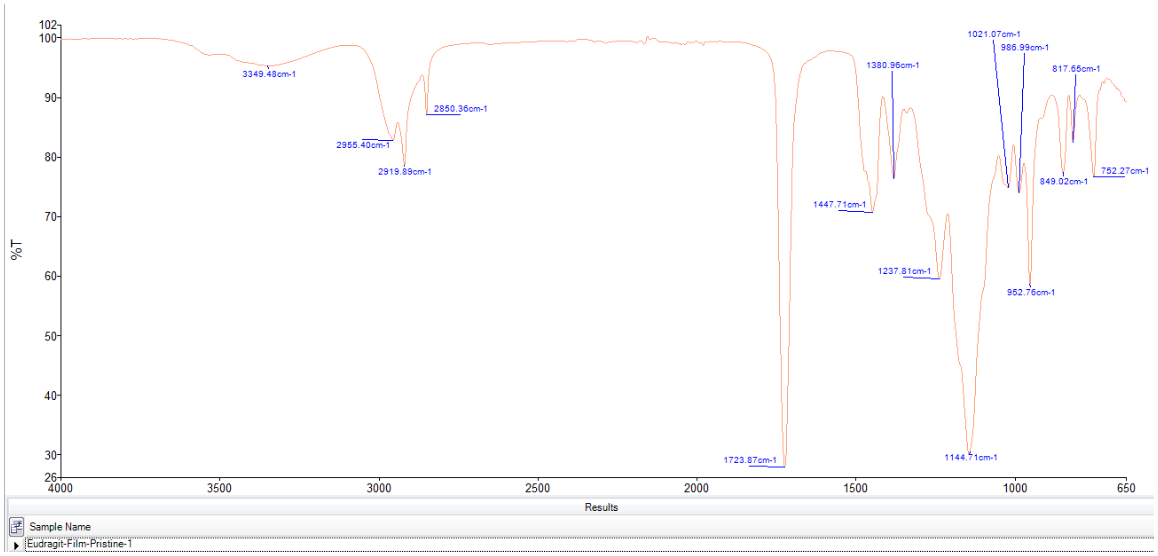

Figure S1. Pristine Eudragit film.

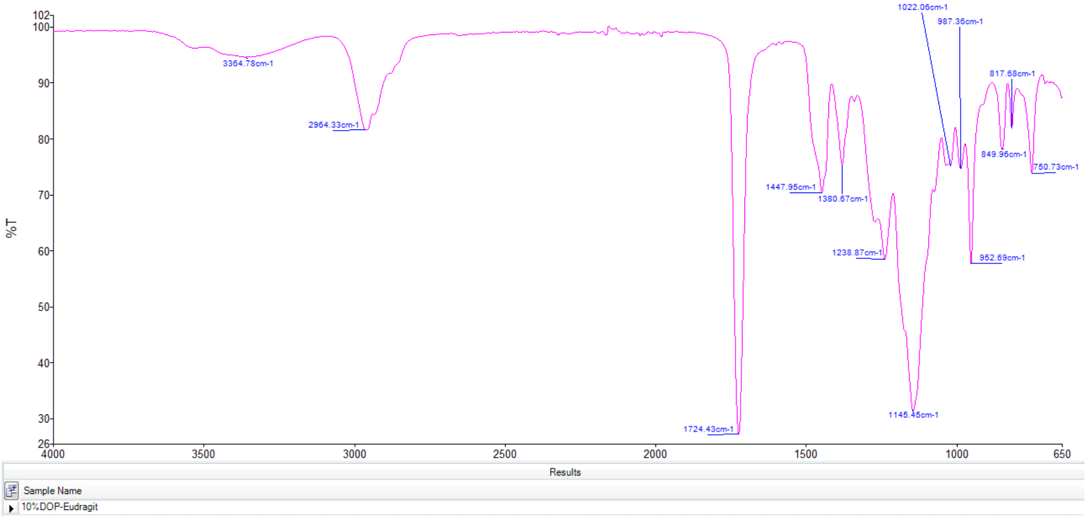

Figure S2. 10% DOP.

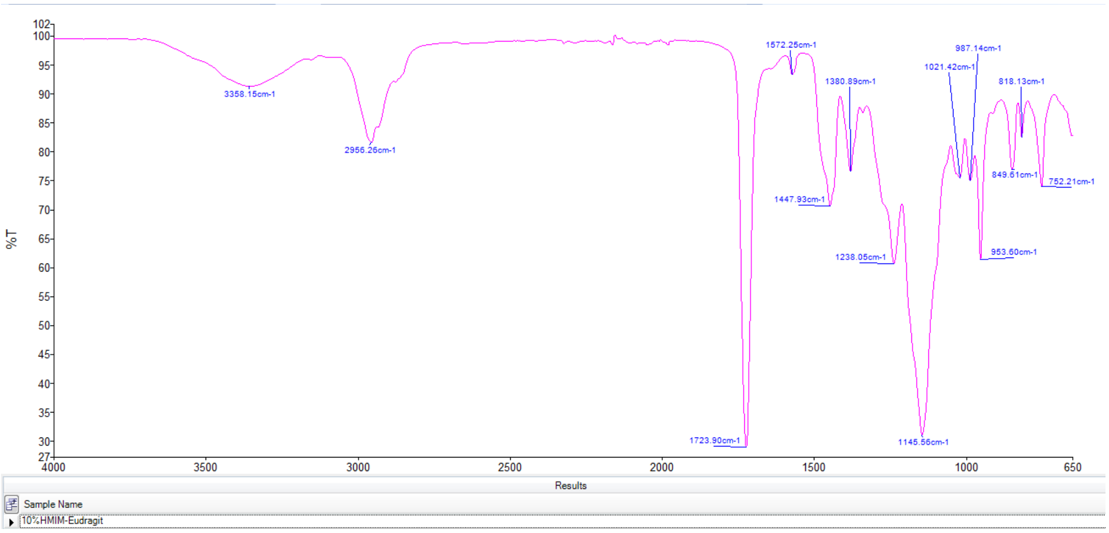

Figure S3. 10% [HMIM]Cl.

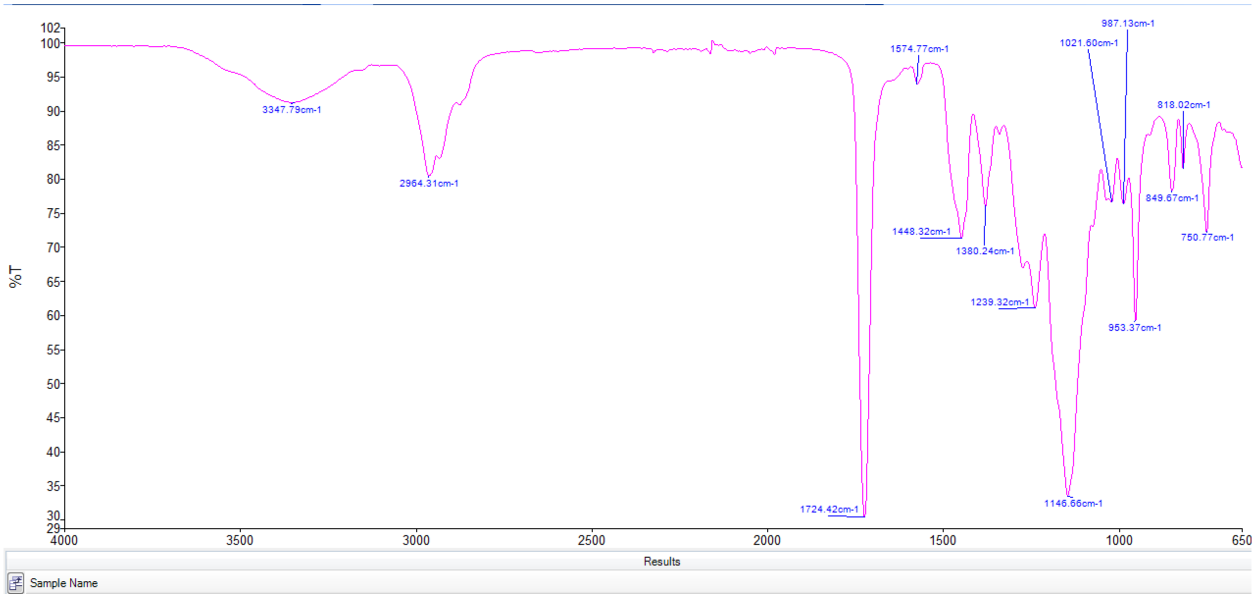

Figure S4. 10% [HMIM]Cl and DOP blend.

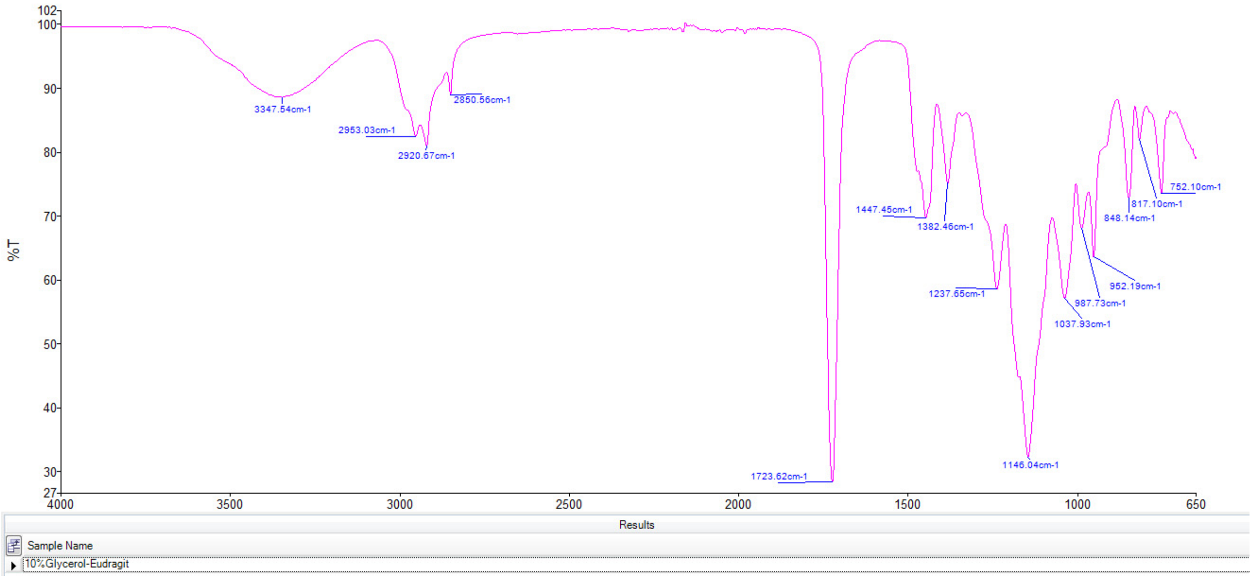

Figure S5. 10% glycerol.
